# Efficacy of *Metarhizium* spp. against gastrointestinal nematodes in goats and evidence of conidia infection through the integument

**DOI:** 10.1590/S1984-296120260010

**Published:** 2026-05-15

**Authors:** Ially de Almeida Moura, Antônio Wesley Oliveira da Silva, Mayara Macêdo Barrozo, Thaís Almeida Corrêa, Patrícia Silva Gôlo, Isabele da Costa Ângelo, Caio Márcio de Oliveira Monteiro, Éverton Kort Kamp Fernandes, Vânia Rita Elias Pinheiro Bittencourt, Alexandre Dias Munhoz, Wendell Marcelo de Souza Perinotto

**Affiliations:** 1 Universidade Estadual de Santa Cruz – UESC, Departamento de Ciências Agrárias e Ambientais, Ilhéus, BA, Brasil; 2 Universidade Federal da Bahia – UFBA, Programa de Ciência Animal nos Trópicos, Salvador, BA, Brasil; 3 Universidade Federal de Goiás – UFG, Departamento de Biociências e Tecnologia do Instituto de Patologia Tropical e Saúde Pública, Goiánia, Goiás, Brasil; 4 Universidade Federal Rural do Rio de Janeiro – UFRRJ, Instituto de Veterinária, Departamento de Parasitologia Animal, Seropédica, RJ, Brasil

**Keywords:** Biological control, entomopathogenic fungi, strongylids, nematophagous fungi, Controle biológico, fungos entomopatogênicos, estrongilídeos, fungos nematófagos

## Abstract

The search for sustainable alternatives to manage gastrointestinal nematodes in small ruminants has intensified due to rising anthelmintic resistance. This study evaluated *in vitro* the efficacy of several Brazilian isolates of the genus *Metarhizium* spp. on gastrointestinal nematodes (GINs) of goats which were naturally infected with mixed populations predominantly composed of *Haemonchus* spp. (63%), *Trichostrongylus* spp. (35%), and *Oesophagostomum* spp. (2%). Biological assays were conducted using quantitative coproculture after 10 days of incubation, and SEM was performed after 72 h of fungus–larva interaction. Isolates LCM S05, LCM S10, LCM S11, LCM S13, LCM S14, and LCM S15 promoted larval reduction of: 50.24%, 43.29%, 44.99%, 55.45%, 52.52%, and 67.95%, respectively. Isolate LCM S15, which showed the greatest reduction in larvae, was tested at concentrations of 1.0 × 10^6^, 1.0 × 10^7^, and 1.0 × 10^8^ conidia/mL, resulting in reductions in larvae of 36.90%, 50.17%, and 68.28%, respectively. In SEM, photomicrographs demonstrated that conidia adhered to and germinated on the nematode integument. This study demonstrates the potential of Brazilian isolates of *Metarhizium* spp. for the control of GINs in goats and highlights the route of fungal infection through the integument.

## Resumo

A busca por alternativas sustentáveis para o manejo de nematóides gastrointestinais em pequenos ruminantes tem se intensificado devido ao aumento da resistência aos anti-helmínticos. Este estudo avaliou *in vitro* a eficácia de vários isolados brasileiros do gênero *Metarhizium* spp. em nematóides gastrointestinais (NGIs) de caprinos naturalmente infectadas com populações mistas compostas predominantemente por *Haemonchus* spp. (63%), *Trichostrongylus* spp. (35%) e *Oesophagostomum* spp. (2%). Os ensaios biológicos foram realizados utilizando coprocultura quantitativa após 10 dias de incubação, e a Microscopia Eletrônica de Varredura (MEV) foi realizada após 72 horas de interação entre o fungo e a larva. Os isolados LCM S05, LCM S10, LCM S11, LCM S13, LCM S14 e LCM S15 promoveram redução no número de larvas: 50,24%, 43,29%, 44,99%, 55,45%, 52,52% e 67,95%, respectivamente. O isolado LCM S15, que apresentou maior redução de larvas, foi testado nas concentrações 1,0 × 10^6^, 1,0 × 10^7^ e 1,0 × 10^8^ conídios/mL, proporcionando reduções de larvas de 36,90%, 50,17%, 68,28%, respectivamente. Na MEV, foi possível constatar nas fotomicrografias que houve aderência e germinação dos conídios no tegumento dos nematoides. O presente estudo demonstra o potencial de isolados brasileiros de *Metarhizium* spp. para o controle de NGIs de caprinos, e evidencia a via de infecção fúngica através do tegumento.

## Introduction

Gastrointestinal helminthiasis is a major health constraint in goat farming (Pires et al., 2020). In northeastern Brazil, goats are commonly infected by nematodes such as *Haemonchus* spp., *Trichostrongylus* spp., *Oesophagostomum* spp., *Trichuris* spp., *Cooperia* spp., and *Strongyloides* spp., which show high prevalence in production systems in the region (Pires et al., 2020; Santos & Clemente, 2025).

Anthelmintic resistance (AR) is an urgent and emerging issue for ruminant production in Brazil, requiring comprehensive assessments of the distribution and use of commercially available drugs, as well as the adoption of effective strategies to slow and prevent its spread (Macedo et al., 2023). The use of ineffective anthelmintics causes significant economic losses, as investments do not translate into therapeutic benefits or improvements in animal health or productivity (Chagas et al., 2022; Ehnert et al., 2025).

Recent studies showed widespread resistance of nematodes to the main drugs available on the market: benzimidazoles, avermectins, imidazothiazoles, salicylanilides, as well as to amino-acetonitrile (monepantel) (Voigt et al., 2022; Soares, 2023; Mavundela & Dzemo 2025). The resistance of nematodes, combined with concerns about the environmental impact of the use of antiparasitics in livestock and their residues in animal products, has driven studies on alternative control methods (Beys-da-Silva et al., 2020; Garcia et al., 2019). In this perspective, biological control emerges as a way to integrate conventional parasite control with the aim of improving the production of small ruminants in livestock farming, ensuring their welfare and reducing impacts on the environment.

Recent studies with *Duddingtonia flagrans* have shown promising results (Fausto et al., 2021; Rodrigues et al., 2021; Rodrigues et al., 2022; Mendes et al., 2023; Nunes et al., 2023). In addition to these promising results, it is important to highlight the recent authorization and commercialization in Brazil of Bioverm®, a fungal formulation based on *D. flagrans*, indicated for the control of gastrointestinal nematodes in ruminants and horses. Studies evaluating this product have demonstrated its effectiveness in reducing infective larvae, reinforcing the potential of *D. flagrans* as a viable biological control agent for gastrointestinal nematodes (Rodrigues et al., 2021). This fungus, like *Clonostachys rosea, Arthrobotrys musiformis*, and *Trichoderma esau,* has been shown to be effective in preying on *H. contortus* larvae (Silva et al., 2017), with being able to reduce dependence on chemical anthelmintics (Jorge-Neto et al., 2025). Despite the advances achieved with nematophagous fungi such as *Duddingtonia flagrans*, there is still considerable scope for research involving other biological agents that may contribute to sustainable parasite control strategies.

*Metarhizium anisopliae* sensu lato (s.l.) is recognized as one of the best candidates for the biological control of a wide variety of arthropods (Pereira et al., 2022; Velázquez-Sarmiento et al., 2024; Miranda et al., 2024), due to its mechanism of action through the integument (Alves, 1998). When it comes to ectoparasite control in ruminants, the fungus *M. anisopliae* s.l. has proven to be highly effective against cattle ticks (Perinotto et al., 2017).

The fungus *M. anisopliae* s.l. has shown potential for controlling free-living stages of gastrointestinal nematodes in horses (Rodrigues et al., 1996) and goats (Moura et al., 2023). However, despite promising results against parasites of cattle and other livestock, the mechanisms by which *Metarhizium* affects gastrointestinal nematodes are still not well understood. In this perspective, the present study aimed to evaluate, through laboratory tests, the efficacy of different Brazilian isolates of *Metarhizium* spp. [viz., *M. anisopliae* (LCM S05), *M. pingshaense* (LCM S10), *M. brunneum* (LCM S11, LCM S13, LCM S14), and *M. robertsii* (LCM S15)] on goat GINs. In addition, the present study also analyzed the adhesion and development of M*. robertsii* LCM S15 conidia on the integument of third-stage (L3) larvae of GINs.

## Material and Methods

### Experimental design

The experiments were conducted under a completely randomized design. Conidial suspensions were prepared by harvesting conidia from fungal cultures and suspending them in sterile aqueous solution containing 0.1% Tween 80, which was used as a surfactant to ensure homogeneous dispersion of the conidia. The control treatment consisted exclusively of the 0.1% Tween 80 solution, allowing the evaluation of fungal effects independent of the dispersant.

In biological assays, isolates of *M. anisopliae* (LCM S05), *M. pingshaense* (LCM S10), *M. brunneum* (LCM S11, LCM S13, LCM S14) and M*. robertsii* (LCM S15) from the Entomopathogenic Fungi Culture Collection of the Microbial Control Laboratory of the Federal Rural University of Rio de Janeiro (UFRRJ).

In the first experiment, the isolates *M. anisopliae* (LCM S05), *M. pingshaense* (LCM S10), *M. brunneum* (LCM S11, LCM S13, LCM S14), and *M. robertsii* (LCM S15) were evaluated in two experimental stages, each comprising three isolates, due to the fungal isolates being obtained at different times. In both stages, the same experimental conditions, design, and evaluation methods were maintained. For each isolate, four experimental groups were established: one control group and three treated groups receiving conidial suspensions at a concentration of 1.0 × 10^8^ conidia/mL, with six replicates per group.

In the second experiment, a dose–response assay was conducted using *M. robertsii* LCM S15. Four experimental groups were formed: a control group and three treated groups exposed to conidial suspensions at concentrations of 1.0 × 10^6^, 1.0 × 10^7^, and 1.0 × 10^8^ conidia/mL, with eight replicates per group.

Treatments were performed by exposing infective larvae (L3) to the respective conidial suspensions under controlled laboratory conditions. After an incubation period of 10 days, larval counts were performed, and treatment efficacy was determined by calculating the percentage of larval reduction, using the following formula:


$$\begin{array}{c} Larval\;reduction\;(\%)=\\ (number\;of\;larvae\;in\;control\;-\;number\;of\;larvae\;in\;treated\;group)/\\ (number\;of\;larvae\;in\;control)\;\times \;100 \end{array}$$ (1)

### Animals used for fecal collection

For the in vitro experiments, fecal samples were collected directly from the rectal ampulla of the animals and analyzed by determining the number of eggs per gram of feces (EPG) using the Gordon and Whitlock (1939) technique. Animals were selected based on fecal samples containing eggs of gastrointestinal nematodes belonging to the superfamilies *Trichostrongyloidea* and *Strongyloidea*, with egg counts ranging from 2,500 to 3,200 EPG.

Thirty-two female crossbred goats aged between six and 24 months and weighing approximately 30 kg on average were used. They belonged to the experimental herd of the Federal University of Recôncavo da Bahia (UFRB) and had not been treated with anthelmintics for at least 60 days.

### *In vitro* biological assays with GINs for characterization and screening of different isolates of *Metarhizium* spp. and concentration testing

The screening of isolates for their ability to reduce larvae was conducted using the quantitative coproculture technique. Fecal samples were collected directly from the rectal ampulla of goats and divided into 2 g aliquots, placed in plastic cups, to which 2 mL of the corresponding fungal suspension (1.0 × 10^8^ conidia/mL) was added. Each treatment was repeated six times. In the control group, instead of using the fungal suspension, 2 mL of 0.1% Tween 80 aqueous solution was used. After 10 minutes, 2 g of wood shavings was added, followed by homogenization of the material. The containers were then covered with perforated plastic film and kept at room temperature for 10 days.

After this period, the cups were filled with water heated to 40 °C and inverted on Petri dishes containing 20 mL of water. After two hours, the material was collected, transferred to test tubes, and analyzed under an optical microscope to identify and quantify the L3 larvae of GINs. The isolate that showed the greatest reduction in the number of larvae was selected for subsequent testing with three different concentrations (1.0 × 10^6^, 1.0 × 10^7^, and 1.0 × 10^8^ conidia/mL), following the same experimental protocol described above, as well as for subsequent tests.

### Evaluation of conidia adhesion and development on the integument of GINs

The larvae used for scanning electron microscopy were randomly selected from the L3 population obtained after coproculture, following a 10-day incubation period. Subsequently, L3 larvae of gastrointestinal nematodes from goats were exposed to *M. robertsii* LCM S15 conidia suspended in an aqueous solution containing 0.1% Tween 80 for 72 hours*.*An aliquot from each group was fixed in 2% paraformaldehyde + 2% glutaraldehyde solution for five days. Subsequently, the fixing solution was completely removed, followed by four washes in PBS buffer (15 minutes each) and immersion in 70% alcohol for 24 hours. The samples were dehydrated in a graduated series of alcohol (80, 90, 95, and 100%) for 30 minutes each and sent to the Multi-user High-Resolution Microscopy Laboratory of the Federal University of Goiás (LABMIC/UFG) for drying and image capture.

After dehydration, the samples were placed in tubes containing hexamethyldisilazane (HMDS) for five minutes. After this period, the excess HMDS was removed, and the Eppendorf tube was left open at room temperature until the reagent had completely evaporated. After drying, the samples were mounted on cylindrical "Stub" sample holders on double-sided copper conductive tape. Subsequently, the samples were coated with a conductive material, gold, using the Desk V Gold Film Deposition System, Denton Vacuum LLC, Moorestown, New Jersey, USA. The samples were then analyzed using a Scanning Electron Microscope (SEM), JSM-6610, Jeol, Tokyo, Japan, equipped with EDS, Thermo Scientific NSS Spectral Imaging, at an acceleration voltage of 8 kV.

### Statistical analysis

The data were initially evaluated for normality using the Shapiro-Wilk test. When the assumptions of normality were met, analysis of variance (ANOVA) was performed, followed by Tukey's test for comparison between means. All statistical analyses were conducted using RStudio software (version 2024.04.2+764).

## Results

### Viability of suspensions

The conidia of *Metarhizium* spp. suspensions used in the experiments showed viability equal to or greater than 99%, determined by the germination rate observed after incubation for 24 hours at 25 ± 1 °C.

### *In vitro* biological assay with GINs

The infective larvae evaluated in the assays originated from goats naturally infected with mixed populations of gastrointestinal nematodes, predominantly *Haemonchus* spp. (63%), *Trichostrongylus* spp. (35%), and *Oesophagostomum* spp (2%).

In the in vitro biological test, conducted in two experimental stages, after 10 days of fungus–larva interaction, in the first test (Table 1), isolates *M. anisopliae* LCM S05, *M. pingshaense* LCM S10, and *M. brunneum* LCM S11 reduced the number of larvae recovered by 50.24%, 43.29%, and 44.99%, respectively. There were no statistical differences between the isolates (p values ranging from 0.6758 to 1.0000), although all differed significantly from the control group (p < 0.001). In the second test (Table 2), the isolates M*. brunneum* (LCM S13 and LCM S14) and M*. robertsii* LCM S15 also showed significant reductions compared to the control, with reductions in the number of larvae recovered of 55.45%, 52.52%, and 67.95%, respectively (p < 0.002). Among the isolates evaluated in this test, no statistical differences were observed (p values ranging from 0.4009 to 0.9899), although M*. robertsii* (LCM S15) showed the greatest reduction in the number of larvae recovered.

**Table 1:** Mean *±* standard deviation of the number of gastrointestinal nematodes larvae recovered after laboratory treatment of goat feces with conidial suspensions of *Metarhizium* isolates*. anisopliae* (LCM S05), *Metarhizium pingshaense* (LCM S10), and *Metarhizium brunneum* (LCM S11), and percentage reduction in the number of larvae in the treatments. The fungus–larva interaction period was 10 days.

Groups	Number of larvae	Reduction of L3 larvae (%)
Control	2772.0±315.9 a	-
LCM S05	1379.3±299.9 b	50.24
LCM S10	1571.8±383.8 b	43.29
LCM S11	1580.1±163.0 b	44.99

Means followed by the same letter in the column do not differ statistically from each other (p ≥ 0.05), according to Tukey's test.

**Table 2:** Mean ± standard deviation of the number of gastrointestinal nematodes larvae recovered from goat feces and subjected to laboratory treatment with conidial suspensions of *Metarhizium brunneum* (LCM S13, LCM S14) and *Metarhizium robertsii* (LCM S15) and percentage reduction of larvae in the treatments. The fungus–larva interaction period was 10 days.

Groups	Number of larvae	Larval reduction (%)
Control	848.1±145.5 a	-
LCM S13	377.8±133.6 b	55.45
LCM S14	402.6±171.9 b	52.52
LCM S15	271.8±88.6 b	67.95

The means followed by the same letter in the column do not differ statistically from each other (p ≥ 0.05), according to Tukey's test.

In a second trial, after 10 days of fungus–larva interaction and using different conidial concentrations, the isolate *M. robertsii* LCM S15 significantly reduced the number of larvae compared to the control group (P < 0.001) with a reduction in L3 larvae ranging from 36.90% to 68.28% (Table 3). The treatments at concentrations of 1.0 ×10^6^ and 1.0 ×10^7^conidia/mL did not ly differ from each other (p = 0.2332), but both had lower means than the control, evidencing the effect of the treatment. The treatment at a concentration of 1.0 × 10^8^ conidia/mL had the lowest mean, differing significantly from the treatment at a concentration of 1.0 × 10^6^ conidia/mL (p = 0.0005) and showing a tendency to differ from the concentration of 1.0 × 10^7^conidia/mL (p = 0.0594). (Table 3).

**Table 3:** Mean *±* standard deviation of gastrointestinal nematodes larvae recovered after laboratory testing with four concentrations of the *Metarhizium robertsii* LCM S15 isolate: 1.0 ×10^6^, 1.0 ×10^7^, and 1.0 ×10^8^ conidia/mL. The fungus–larva interaction period was 10 days.

Groups	Number of larvae	Larval reduction (%)
Control	574.7±118.5 a	-
1.0 × 10^6^	363.3±81.6 b	36.90
1.0 × 10^7^	286.9±30.5 bc	50.17
1.0 × 10^8^	182.6±64.1 c	68.28

The means followed by the same letter in the column do not differ statistically from each other (p ≥ 0.05), according to Tukey's test.

### Evaluation of conidia adhesion and development on the integument of L3 larvae of GINs

**Figure 1:**
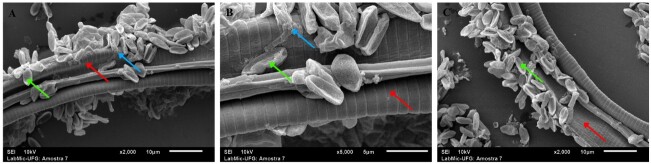
Scanning electron micrographs of *Metarhizium robertsii* LCM S15 interacting with goat gastrointestinal nematodes. (A–C) Scanning electron microscopy images show conidia (green arrows) adhering to the nematode cuticle; the digestive tract, indicating conidia germination (blue arrows); and the nematode cuticle (red arrows), highlighted as the nematode's main structural barrier against fungal infection. Images A and C obtained at 2000× magnification; image B at 5000× magnification.

In SEM analysis, it was possible to observe the adhesion and germination of *M. robertsii* conidia on the integument of L3 larvae of GINs 72 hours after exposure to the aqueous fungal suspension of *M. robertsii* (LCM S15) (Figure 1).

## Discussion

This fungus has already demonstrated the ability to infect different insect species, such as *Aedes aegypti* (Rodrigues et al., 2019), *Lutzomyia longipalpis* (Diptera: Psychodidae) (Amóra et al., 2010), as well as several species of ticks (Perinotto et al., 2017; Marciano et al., 2020; Barbieri et al., 2023).

In the present study, variation was found in the number of larvae recovered from the six isolates evaluated. Although all isolates promoted a reduction in the number of nematode larvae, the highlight was the isolate *M. robertsii* LCM S15, which presented the highest reduction rate in the number of larvae recovered after treatment (67.95%). These findings are consistent with those of Moura et al. (2023), who also observed significant differences between groups treated with *M. anisopliae* s.l. and control groups in laboratory trials, confirming the action of the fungus on GINs. This is the only study that investigated the action of *M. anisopliae* against goat helminths, reinforcing the need for further research in this area.

The highest conidial concentration of the isolate *M. robertsii* LCM S15 (1.0 × 10^8^ conidia/mL) was more effective than the lower concentrations, corroborating the results of Rodrigues et al. (1996), who demonstrated a greater effect of *M. anisopliae* s.l. suspensions at a concentration of 1.3 × 10^8^ compared to 1.3 × 10^6^ on Cyathostominae larvae in horses, with reductions between 55.4% and 85.1%. Although there are few studies evaluating the action of entomopathogenic fungi specifically against GINs, recent research with nematophagous fungi *Duddingtonia flagrans* has demonstrated in *in vitro* trials that efficacy depends directly on the concentration of chlamydospores applied (Paoletti et al., 2024). Similarly, Nunes et al. (2023) reported that a commercial formulation containing *D. flagrans,* at concentrations of 0.4g and 1g, was capable of reducing the recovery of larvae in equine feces by 44.23% and 57.2%, respectively, after 10 days of incubation under controlled laboratory conditions, reinforcing that the concentration of the fungus directly impacts the reduction of larvae.

These findings reinforce the importance of comparative studies that evaluate different concentrations of fungal suspensions, since, although *Metarhizium* spp. is easy to produce on a large scale (Orlandelli & Pamphile, 2011), it is essential to consider the cost-benefit balance. Although, after 10 days of fungus–larva interaction, the concentration of 1.0 ×10^8^ showed the greatest reduction in the number of L3 larvae, the results indicate that 1.0 ×10^7^ conidia/mL is an option that may offer a better cost-benefit ratio.

Despite these findings, it is important to emphasize that the concentrations evaluated were tested exclusively under in vitro conditions, and no specific delivery method is proposed at this stage. Although biological control using fungi is recognized as an important component of integrated and sustainable pest management (Mesquita et al., 2023), the practical application of *Metarhizium* spp. in livestock systems involves challenges related to interaction with fecal matter, environmental exposure, and application efficiency.

In the context of biological control of GINs, nematophagous fungi such as *D. flagrans, C. rosea, A. musiformis*, and *T. esau* (Silva et al., 2017), as well as *Arthrobotrys oligospora, Dactylaria scaphoides*, and *Nematoctonus leiosporus* (Sander & Neumann, 2025), have already demonstrated their ability to capture and destroy nematode larvae using three-dimensional traps. However, the mechanism of action of these fungi differs from that observed in entomopathogenic fungi, such as *M. anisopliae*, in which conidia germinate on the integument of ticks, develop germ tubes, and penetrate the body, multiplying in the form of hyphae until the host dies (Arruda et al., 2005).

In the case of *M. anisopliae*, specific mechanisms of action on GINs have not yet been described. However, its infection process has been well characterized in ticks, where conidia adhere to the host surface, germinate on the external cuticle, and form germ tubes that penetrate the integument. Once inside the host, the fungus proliferates as hyphae, ultimately leading to host death. (Arruda et al., 2005), and recent studies have shown that hydrophobic proteins, such as hydrophobins, present in the conidia wall, promote efficient adhesion even in aqueous environments, allowing the infectious process to begin (Greenfield et al., 2014). In the present study, the tests were conducted over a period of 72 hours, defined based on a previous study that indicated this interval as the most appropriate for observing the activity of the fungus on the larvae, and it was evident that conidia of *M. robertsii* LCM S15 were able to adhere to the integument, as well as germinate and form the germ tube penetration of the cuticle towards the underlying tissues of goat GINs. From a biological perspective, the results support the hypothesis that *Metarhizium spp.* may act on gastrointestinal nematodes through early infection events similar to those described for arthropod hosts. The observed adhesion of conidia to the larval integument, followed by germination and germ tube formation, indicates that the nematode cuticle can serve as a substrate for fungal attachment and initial colonization. Although larval degradation was not evaluated, these early steps are considered essential prerequisites for subsequent penetration and pathogenic development. This is a fundamental step in understanding the mechanism of action of *Metarhizium* spp. on goat GINs and other animals.

Studies of SEM in ruminant GINs have already shown that conidia penetrate the host through the production of enzymes, especially proteases of the Pr1 and aminopeptidase families, which degrade the parasite's integument (Aw & Hue, 2017). The integument of nematodes consists of a predominantly collagenous extracellular matrix secreted by the hypodermis, a layer of ectodermal cells that covers the entire body of the animal. This structure functions as an exoskeleton and resembles that of other parasites (Page & Johnstone, 2007). It can therefore be inferred that this enzymatic process, already demonstrated in other parasites, may occur in helminths in a very similar way.

To date, there is no evidence that GINs have mechanisms that directly inhibit the development and penetration of *Metarhizium* spp. However, studies with ticks such as *Amblyomma sculptum* and *Dermacentor nitens* show that the integument of these arthropods contains lipid components capable of hindering fungal penetration (Ribeiro-Silva et al., 2022). In addition, it has been observed that mild fungal infections can be partially controlled by the immune response of ticks, but mortality increases when phagocytosis of conidia by hemocytes is compromised (Fiorotti et al., 2022).

In practical terms, the use of *Metarhizium* spp. against GINs is expected to complement existing parasite control strategies by targeting free-living larval stages in fecal substrates and the environment. Its scalability of production and low toxicological risk to mammals, birds, fish, and invertebrates (Zimmermann, 2007), support its potential inclusion in integrated parasite management programs. However, further *in vivo* and field studies are required to define formulation strategies, delivery methods, and efficacy under grazing conditions.

Based on the results found, it can be concluded that this study provides data on six Brazilian isolates of *Metarhizium* spp. against GINs and highlights the route of fungal infection through the integument. This important contribution points to future investigations that may select *Metarhizium* spp. as a promising biological control agent for gastrointestinal nematodes in production animals.

## Data Availability

The datasets generated and/or analyzed during the current study are available from the corresponding author.
